# Clinical and epidemiological profiles of individuals with drug-resistant tuberculosis

**DOI:** 10.1590/0074-02760140316

**Published:** 2015-04

**Authors:** Heloisa da Silveira Paro Pedro, Susilene Maria Tonelli Nardi, Maria Izabel Ferreira Pereira, Rosângela Siqueira Oliveira, Philip Noel Suffys, Harrison Magdinier Gomes, Amanda Juliane Finardi, Eloise Brasil de Moraes, Ida Maria Foschiani Dias Baptista, Ricardo Luiz Dantas Machado, Lilian Castiglioni

**Affiliations:** 1 Núcleo de Ciências Biomédicas, Centro de Laboratório Regional de São José do Rio Preto, Instituto Adolfo Lutz, São José do Rio Preto, SP, Brasil; 2 Departamento de Genética, Universidade Estadual Paulista Júlio de Mesquita Filho, São José do Rio Preto, SP, Brasil; 3 Núcleo de Tuberculose e Micobacterioses, Instituto Adolfo Lutz Central, São Paulo, SP, Brasil; 4 Laboratório de Biologia Molecular aplicada a Micobactérias, Fundação Oswaldo Cruz, Rio de Janeiro, RJ, Brasil; 5 Divisão de Pesquisa e Ensino, Instituto Lauro de Souza Lima, Bauru, SP, Brasil; 6 Departamento de Parasitologia, Instituto Evandro Chagas, Serviço de Vigilância em Saúde, Ministério da Saúde, Belém, PA, Brasil; 7 Departamento de Epidemiologia e Saúde Coletiva, Faculdade de Medicina de São José do Rio Preto, São José do Rio Preto, SP, Brasil; 8 Departamento de Biologia, Centro Universitário de São José do Rio Preto, São José do Rio Preto, SP, Brasil

**Keywords:** tuberculosis, diagnosis, antimicrobial resistance

## Abstract

Drug-resistant tuberculosis (TB) is a growing global threat. Approximately 450,000 people developed multidrug-resistant TB worldwide in 2012 and an estimated 170,000 people died from the disease. This paper describes the sociodemographic, clinical-epidemiological and bacteriological aspects of TB and correlates these features with the distribution of anti-TB drug resistance. Mycobacterium tuberculosis (MT) cultures and drug susceptibility testing were performed according to the BACTEC MGIT 960 method. The results demonstrated that MT strains from individuals who received treatment for TB and people who were infected with human immunodeficiency virus were more resistant to TB drugs compared to other individuals (p < 0.05). Approximately half of the individuals received supervised treatment, but most drug-resistant cases were positive for pulmonary TB and exhibited positive acid-fast bacilli smears, which are complicating factors for TB control programs. Primary healthcare is the ideal level for early disease detection, but tertiary healthcare is the most common entry point for patients into the system. These factors require special attention from healthcare managers and professionals to effectively control and monitor the spread of TB drug-resistant cases.

An inadequate capacity to quickly and accurately diagnose tuberculosis (TB) in developing countries (WHO 2011 ) and the growing threat of multidrug-resistant TB *strains* (MDR-TB) are global concerns (Said et al. 2012). Approximately 450,000 people developed MDR-TB worldwide in 2012, with an estimated 170,000 deaths annually. Most TB cases and deaths occurred in men, but this disease remains one of the top three causes of deaths in women worldwide (WHO 2013). The lack of low-cost testing, the long duration of treatment, the lack of an effective vaccine and the emergence of drug-resistant TB in low-income countries are limiting factors for disease control worldwide (Lawn & Zumla 2011).

The Brazilian National Tuberculosis Control Program (PNCT) recommends antibiotic susceptibility testing (AST) to evaluate bacilli resistance to anti-TB drugs, detect treatment failure and monitor the primary and/or acquired nature of resistance, which interrupts the transmission cycle of drug *resistance *to control and cure the disease (MS/SVS/DVE 2008, 2011, Parrish & Carroll 2011). These strategies reduce the risk of drug-resistant TB and MDR-TB, which are particularly important in immunosuppressed individuals. AST is performed using the following first-line drugs: streptomycin (SM), rifampicin (RMP), isoniazid (INH), ethambutol (EMB) and pyrazinamide (PZA) (MS/SVS/DVE 2011).

Epidemiological features and disease control vary according to the geographic region. Therefore, knowledge of the characteristics of patients with drug-resistant TB is essential to develop therapeutic measures and establish policies to control the problem in Brazil (Melo et al. 2003).

This study identified the antimicrobial response of MT to the drugs that comprise anti-TB polychemotherapy to identify possible correlations between resistance and the sociodemographic and clinical-epidemiological features of patients.

## MATERIALS AND METHODS

This cross-sectional study was conducted in a city located in the northern region of the state of São Paulo (SP) (coordinates: 20º49'11''S 49º22'46''W) with a population of 408,258 individuals (IBGE 2010) [estimated population in 2014: 415,769 individuals (SES/CVE/DCT 2008) ]. This municipality is a priority in the PNCT because of the high incidences of TB and human immunodeficiency virus (HIV) (SES/CVE/DCT 2008, Vendramini et al. 2010, SMPGE 2013).

The majority of the population in this study lives in a city with demographic and social indicators that are similar to developed countries. The United Nations Development Program considers a Human Development Index of 0.797 high and the levels of wealth, life expectancy and education are good (SMPGE 2013). The incidence of TB in 2009 was 23.1/100,000. The TB-HIV co-infection rate was 23% and the TB detection rate was 73.2%. Cure, dropout and mortality rates were 77.7%, 6.8% and 15.5%, respectively (SMSH 2009, Ponce et al. 2013).

The population of this study consisted of patients whose samples were cultured, identified and subjected to AST of MT between 2009-2013. Patients of both genders who were suspected of having TB that was subsequently confirmed using a positive acid-fast bacilli (AFB) smear or MT strain typing were included in the study*.*

Data were retrieved from the records of the regional reference laboratory for the Epidemiological Surveillance Groups (GVE) 29 and 30, which serve 102 municipalities in the region and an estimated population of 1,493,835 inhabitants [population of GVE 29: 1,237,177 and population of GVE 30: 256,658 (SES/CVE/DCT 2008)]. All cultures from GVE 30 were sent to the reference laboratory. Other laboratories also (2 hospitals and 3 laboratories from the private healthcare network) cultured samples from GVE 29. However, culture samples that were found positive in other laboratories and fulfilled the criteria established by the PNCT (SES-SP/CVE/DT 2002, MS/SVS/DVE 2008) were sent to the reference laboratory for typing and susceptibility testing.

Sociodemographic and clinical-epidemiological data were collected from the medical records of patients who received TB treatment in the Municipal Reference Centre for Tuberculosis Control and Government Health Clinics and were complemented by the TB Surveillance System of São Paulo State (TB-WEB) (SES/CCD/CVE/DCT 2008), which is a system that was developed as a joint venture between the State Department of Health and São Paulo State Data Processing Company. Collected data included gender, age, years of schooling, AFB smear and HIV test results, deprivation of liberty, use of alcohol, tobacco and illicit drugs, clinical presentation of the disease and details of previous TB treatment.

All subjects with positive MT complex samples for which AST was performed in the mycobacteria laboratory of the Regional Reference Laboratory during the study period were included.

Samples that were contaminated during strain identification or AST and samples with insufficient material to perform all tests were excluded.

Samples in the laboratory were used for the direct examination of AFB in smears stained using the Ziehl-Neelsen method. Bacilli were counted using a semiquantitative scale (score) according to the bacterial index as recommended by the Brazilian Ministry of Health (MS/SVS/DVE 2008).

Associated microbiota were eliminated from clinical specimens during culturing following the neutralisation and decontamination procedures of Petroff's method. The decontaminated material was inoculated in flasks containing MGIT 960 medium (Becton & Dickinson, USA) according to the manufacturer's instructions (Parrish & Carroll 2011).

Positive cultures were examined using AFB after subculture in Ogawa-Kudoh medium in a bacteriological incubator at 37ºC to obtain colonies (Miller et al. 2002). MT was identified in grown colonies using the PNB growth inhibition method (medium with p-nitrobenzoic acid at 500 µg/mL) to identify colony morphology and confirm the presence of cord factor following the techniques recommended in the Manual of Tuberculosis Bacteriology (MS/SVS/DVE 2008).

Strains identified as MT were tested to assess their susceptibility to the antimicrobial drugs that comprise the polychemotherapy recommended by the Brazilian Ministry of Health: INH, RMP, SM and EMB (MS/SVS/DVE 2008). Susceptibility to PZA was performed separately using the pyrazinamidase test (Collins et al. 1997).

Susceptibility testing was performed using the BACTEC MGIT 960 system (Becton & Dickinson) following the manufacturer's instructions with some modifications as suggested by Giampaglia et al. (2007). Briefly, the turbidity of the MT inoculum was adjusted to McFarland scale 1. This suspension was diluted 1:5 and 1:100 using sterile distilled water. Then, 0.5 mL of the 1:5 inoculum was added to tubes containing BBL-MGIT 960 medium and the following drugs in the respective final concentrations were added: SM 1.0 µg/mL, INH 0.1 µg/mL, RMP 1.0 µg/mL and EMB 5.0 µg/mL. Simultaneously, the 1:100 suspension was used to inoculate a tube containing the culture medium without drugs (growth control tube).

The tubes were incubated for five and 12 days at 37ºC in an automated MGIT 960 device (Becton & Dickinson). Readings were taken at hourly intervals during this period until the final growth index was attained in the growth control tube and a subsequent final report was issued (Kruuner et al. 2006, Rüsch-Gerdes et al. 2006, Parrish & Carroll 2011).

Patients resistant to at least one antibiotic in the polychemotherapy regimen for TB were classified as mono-resistant. Patients resistant to at least INH and RMP based on the AST were classified as MDR-TB and patients with resistance to two or more drugs, except for both RMP and INH, were classified as poly-resistant (MS/SVS/DVE 2011).

Patients were stratified based on AFB smear results (positive or negative), schooling (less than, equal to or more than 8 years of education) and age (15-45 years and older than 45 years) for statistical analyses.

Chi-square and Fisher's exact tests were used for statistical analyses and the odds ratio was used as a measure of association. A significance level of 5% was adopted.

The Research Ethical Committee of the Adolfo Lutz Institute in SP approved the study (protocol 079/2011).

## RESULTS

The TB-WEB database identified 1,579 patients who received TB treatment in GVE 29 and 30 during the study period (municipal: 794 subjects; regional: 785 subjects). AFB smears (n = 887) and cultures (n = 151) were used as the diagnostic criteria for 1,038 of the patients registered in the TB-WEB system. There was no bacteriological confirmation for the remaining cases. The population of the current study included 84.1% (n = 127/151) of TB cases confirmed by cultures and 24.9% (n = 221/887) of cases confirmed by AFB smears.

A total of 348 MT strains, isolated from 348 individuals, were subjected to AST. All of these patients were being treated in the Healthcare Services of the region.

The patients had a mean age of 38.8 years [standard deviation (SD) 3.6] and the majority (n = 295; 84.8%) were male. Stratification according to the TB-WEB system revealed that most patients had either four-seven years (36.4%) or eight-11 years (28.9%) of schooling, 18.4% of subjects were diagnosed with TB previously and 18.4% were HIV-positive. Alcoholism (27.6%), smoking (20.4%) and drug addiction (17%) were the other most commonly observed problems. Approximately 94% of cases were positive for pulmonary TB and 56% received supervised treatment or directly observed treatment, short-course.

Collected clinical samples sent to the laboratory included sputum (95.6%), bronchoalveolar lavage fluid (1.4%), gastric lavage fluid (1.2%), pleural fluid (0.6%), blood (0.6%), ascitic fluid (0.3%) and cerebrospinal fluid (0.3%). A total of 70.1% of the samples were used to diagnose TB and 29.9% were used for treatment control. The highest number of MT strains (33.6%) was identified in prisons, followed by primary (28.2%), tertiary (24.1%) and secondary healthcare institutions (12.9%). The lowest percentage of MT strains (1.2%) was identified in the private healthcare network.

A total of 317/348 (91.1%) of the analysed cases were susceptible to all tested drugs and 31/348 (8.9%) were resistant to one or more drugs used in TB treatment regimens. Drug-resistance was distributed as follows: 23 strains were resistant to one drug used to treat TB; MDR-TB was observed in six strains and poly-resistance was observed in two strains (Table I).

**TABLE I:** Laboratory diagnostic results of clinical specimens from patients diagnosed with Mycobacterium tuberculosis^a^

Tests	n (%)
Acid-fast bacilli smear	
Positive	221 (63.5)
Negative	127 (36.5)
Antibiotic susceptibility testing	
Susceptible	317 (91.1)
Resistant	
Mono-resistant (n = 23)	
INH	7 (2)
RMP	4 (1.1)
PZA	-
SM	12 (3.4)
EMB	-
Multidrug-resistant (n = 6)	
INH + RMP INH + RMP + PZA SM + INH + PZA + RMP	2 (0.6) 3 (0.9) 1 (0.3)
Poly-resistant (n = 2)	
SM + INH	2 (0.6)
Total	31 (8.9)

a: n = 348; EMB: ethambutol; INH: isoniazid; PZA: pyrazinamide; RMP: rifampicin; SM: streptomycin.

Seven of the patients with any type of resistant strain were diagnosed at the primary healthcare level and 10 patients were diagnosed at the secondary healthcare level. Nine patients were diagnosed at the tertiary healthcare level. Three patients were diagnosed in closed institutions (prisons) and two patients were diagnosed in other institutions. AST was performed concomitant to TB diagnostic tests for 24 patients with resistant strains*.* All 31 MT isolates were obtained from pulmonary clinical specimens.

Table II describes the distribution of sociodemographic, epidemiological and clinical aspects associated with drug resistance. Patients most frequently appeared in the 15-45 year age group, who had less than eight years of schooling, with smear-positive pulmonary TB and who submitted to supervised treatment. Other features, such as the use of alcohol and tobacco and being deprived of liberty, were not positively associated with TB resistance.

Drug-resistant TB was positively associated with HIV infection and previous TB treatment (Table II).

**TABLE II:** Distribution of the main sociodemographic, epidemiological and clinical characteristics associated with drug resistance

Characteristic	Susceptible	Resistant	OR	95% CI	p	Total^*a*^
Gender						
Male	269	26	0.92	0.33-2.53	0.900	295
Female	48	5				53
						348
Age group (years)						
15-45	230	23	0.91	0.39-2.13	0.987	253
> 45	87	8				95
						348
Educational level^*a *^(years)						
< 8	164	12	1.04	0.39-2.64	1.000	176
≥ 8	105	8				113
						289^*a*^
HIV^*a*^						
Negative	248	17	3.36	1.51-7.47	0.004	265
Positive	52	12				64
						329^*a*^
AFB smear						
Negative	117	10	1.22	0.55-2.69	0.698	127
Positive	200	21				221
						348
Deprivation of liberty^*a*^						
No	174	21	0.33	0.12-0.91	0.035	191
Yes	124	5				129
						320^*a*^
Alcoholism^*a*^						
No	225	20	1.30	0.58-2.90	0.526	245
Yes	86	10				96
						341^*a*^
Tobacco smoking^*a*^						
No	246	23	1.16	0.48-2.84	0.813	269
Yes	64	7				71
						340^*a*^
Drug addiction^*a*^						
No	256	28	0.32	0.07-1.41	0.133	284
Yes	56	2				58
						342^*a*^
Clinical presentation						
Pulmonary	304	31	2.66	0.00-2.66	0.165	335
Extrapulmonary	13	-				13
						348
Previous TB treatment^*a*^						
No	259	14	5.66	2.57-12.47	< 0.001	273
Yes	49	15				64
						337^*a*^
Type of treatment^*a*^						
Supervised	180	15	0.79	0.31-2.00	0.818	195
Self-administered	106	7				113
						308^*a*^

a: data not available at TB Surveillance System of São Paulo State; AFB: acid-fast bacilli; CI: confidence interval; HIV: human immunodeficiency virus; OR: odds ratio; TB: tuberculosis.

Seven (7/31) patients with drug-resistant TB were also HIV-positive and received previous treatment. The most frequent resistance profile in these individuals was INH-mono-resistant TB (n = 3).

## DISCUSSION

A further understanding of drug-resistant TB is essential to eradicate this disease worldwide. This study demonstrated the resistance of MT strains to TB polychemotherapy in a strategic region of SP. The rate of drug-resistance in the study area was lower than other parts of the country, but the study area lies where SP converges with the southern region of the states of Minas Gerais (MG) and Mato Grosso do Sul. The study area is the headquarters of the eighth São Paulo Administrative Region, which is an important centre for healthcare and other services (SMPGE 2013). Healthcare services are provided to the local population and the region of the states mentioned above and the state of Goiás (Gazetta et al. 2007). This region is also a distribution centre of illicit drugs to other states and it has a high rate of acquired immune deficiency syndrome (AIDS), which influences TB co-infections (Santos et al. 2009). Control measures need to be prioritised to contain the spread of resistant strains because of the geographical proximity to other Brazilian states and the intense migration of people seeking jobs.

The results of gender (Fandinho et al. 1999, Brito et al. 2004, Zaman et al. 2005, Mendes et al. 2007, Rozman et al. 2007, Vieira et al. 2007, Coelho et al. 2012, Micheletti et al. 2014), mean age (Teixeira et al. 2001, de Souza et al. 2006, Mendes et al. 2007, Rozman et al. 2007, Vieira et al. 2007, Marques et al. 2010, Coelho et al. 2012, Micheletti et al. 2014) and educational level (Brito et al. 2004, Vieira et al. 2007) were similar to other authors. These data demonstrate that TB mainly affects adult males with low educational levels (Baptista et al. 2002, Melo et al. 2003, Motta et al. 2009, Vendramini et al. 2010, Valença et al. 2012).

Co-infection with HIV is a challenge for the diagnosis and treatment of TB and disease control requires a global effort (Lawn & Zumla 2011, MS/SVS/DVE 2011, Müller et al. 2013, Zumla et al. 2013). The municipality in this study exhibited high co-infection rates compared to national and state levels (Vendramini et al. 2010). Therefore, this area is a priority for TB control. Anti-TB drug resistance was associated with HIV infection in this study. Approximately 40% of the drug-resistant strains were from patients living with HIV/AIDS, which suggests that special attention and healthcare measures should be targeted to this population. The World Health Organization (WHO) recommends that these patients undergo AST at the beginning of treatment and the standard WHO-recommended drug treatment should be adopted while awaiting the AST results. Rapid molecular tests should also be used to detect drug-resistant TB (CT/GTD 2009).

Most drug-resistant TB cases occur because of irregular treatment and treatment dropout. The current study demonstrated that previous TB treatment was associated with drug resistance, which was described previously (de Souza et al. 2006, Faustini et al. 2006, Hoffner et al. 2009, Wang et al. 2012, Al-Hajoj et al. 2013, Liu et al. 2013, Micheletti et al. 2014). Other factors, such as alcoholism and illicit drugs, were not associated with antibiotic resistance, but these factors contribute to the treatment dropout rate and noncompliance to treatment (MS/SVS/DVE 2011). Additionally, interactions with antiretroviral therapy may cause intolerance and toxic effects (Zumla et al. 2013). Smoking may also prolong treatment and overload the public healthcare system because smoking is associated with delayed sputum culture conversion and a higher recurrence rate (CT/GTD 2009). The economic impact should also be mentioned because the treatment of drug-resistant cases is more expensive than nondrug-resistant cases (García 2003, de Souza et al. 2006). Drug-resistant TB is more difficult to treat because it may prolong the infectious period, reduce the odds of cure and result in increased transmission (French et al. 2008).

The high number of patients with resistant strains who exhibited positive AFB smears contributes to disease transmission. Notably, the percentage of patients with resistant strains at diagnosis may facilitate the spread of resistant MT, which could be a complicating factor for TB control programs and therapeutic regimens. These results suggest the need for a systematic monitoring of circulating strains. Approximately half of the individuals underwent supervised treatment, but most individuals had pulmonary cases that were used for diagnostic assessment. These cases were smear-positive and drug-resistant, which are complicating factors for control programs and require the attention of surveillance teams with rapid interventions, as stressed by the WHO (2011). This type of surveillance is essential to effectively monitor and control the spread of a global TB epidemic (Talip et al. 2013) and reduce the transmission of resistant strains (Marais et al. 2013).

The local healthcare network is composed of 13 Government Healthcare Clinics, 11 Family Healthcare Clinics, five Emergency Care Clinics, one Tuberculosis and Leprosy Outpatient Clinic, one Sexually Transmitted Disease/AIDS Outpatient Clinic and six hospitals (Ponce et al. 2013). However, more than half of the cases in this study were diagnosed at the tertiary level (i.e., in hospitals where the patient is usually diagnosed in a severe condition) or prisons, which are closed institutions with a high risk of TB transmission. Ponce et al. (2013) reported that approximately 40% of the cases were diagnosed at hospitals since 2000, which was after the decentralisation of TB control measures and investments in primary healthcare. The rate of total TB cases and drug-resistant cases in the current study (24.1%) was lower than the abovementioned study. However, MDR-TB (SM+INH+PZA+RMP) was detected in a young female healthcare professional who died quickly during the early treatment stage of the disease. The fact that she was a healthcare professional at a hospital is worrisome because there was a risk of transmission/exposure of this resistant strain to other professionals and patients of that hospital. The Centre for Epidemiological Surveillance (SES/CVE/DCT 2013) recommends that all TB cases in healthcare professionals be investigated quickly. Surveillance, hospital infection control and occupational healthcare services should be involved in these investigations because the Social Welfare Regulation of the Brazilian Ministry of Health (MS/SVS 1999) considers TB an occupational or work-related disease. Primary healthcare is the ideal level for early disease detection, but tertiary healthcare has been the entry point of patients into the system, which increases the risk of transmission and suggests that TB diagnosis is delayed. This fact requires special attention from healthcare managers and professionals.

Prisoners are at a greater risk of infections because of conditions that include overcrowding, poor air circulation, poor hygiene and sanitation, nutritional deficiency, high-risk behaviours, including alcohol abuse and illicit drug use and contact with other infectious diseases (Martin et al. 1994, Reyes & Coninx 1997, Lobacheva et al. 2007).

Notably, the results of this study indicate that resistance to anti-TB antibiotics was lower in liberty-deprived individuals than nondetainees. However, the WHO recommends that TB control policies should target prisons and long-term care facilities to minimise the risk of transmission by instituting changes in the organisation of the service, including training and a reorganisation of care. Local TB control programs should prioritise health surveillance measures to diagnose the disease at the primary healthcare level (MS/SVS/DVE 2011). Additionally, professionals working in governmental and nongovernmental institutions should discuss the need for more investment targeted at groups who are at highest risk for resistant MT strains (e.g., HIV-positive individuals, individuals in contact with patients with drug-resistant TB, homeless people and patients seen in hospitals and prisons without effective control measures) (Kritski 2010).

Studies in different regions of the states of Rio de Janeiro, Rio Grande do Sul, Ceará, Mato Grosso, SP and MG reported variations in resistance levels in respiratory samples (Araújo et al. 2005, de Souza et al. 2006, Mendes et al. 2007, Rozman et al. 2007, Marques et al. 2010, Coelho et al. 2012, Micheletti et al. 2014). Resistance to any drug in the current study was detected in 8.9% of the cases, which is lower than the abovementioned values. MDR-TB cases are less common, but these cases are the most severe (Pereira 2012). The rates of MDR and poly-resistance in the current study were also identified in other studies in the state (Baptista et al. 2002, Rozman et al. 2007, Coelho et al. 2012).

The lack of complete information of some variables in the TB-WEB system hindered analyses in this study. Nevertheless, the information presented here is valuable for the management and prevention of drug-resistant TB in the study area and other areas of the country.

It is important to emphasise that knowledge of the molecular profiles of the strains that are circulating in the region and the implementation of rapid molecular tests for *routine diagnosis* will promote faster measures to control TB, particularly the transmission of TB strains that are resistant to currently available treatment drugs. Skills and resources are required for these tests, which are in accordance with the Stop TB Strategy towards eradication. These tests should be implemented particularly in developing countries, such as Brazil (Zumla et al. 2013).

The sociodemographic and clinical-epidemiological features of infections caused by MT revealed that most TB patients were working-age adult males with less than eight years of schooling. Pulmonary TB accounted for the majority of TB cases and more than half of the patients received supervised treatment. TB was diagnosed primarily in closed institutions or at the tertiary healthcare level.

High rates of resistance to TB treatment drugs were detected in individuals who were HIV-positive or who had previously received TB treatment. Alcoholism, smoking and drug addiction were not correlated with drug resistance. Resistance to anti-TB antibiotics was lower in liberty-deprived individuals than nondetainees.

The status of the antimicrobial response of MT to anti-TB polychemotherapy revealed that most of these patients were diagnosed at secondary and tertiary healthcare levels and closed institutions. The frequency of mono-resistant strains was higher in these patients, but the presence of patients with MDR-TB in hospitals requires closer attention.

The majority of patients with resistant strains had positive smears, which is a high risk factor for disease transmission that facilitates the spread of resistant MT strains.
